# Incidental unilateral idiopathic maculopathy in children

**DOI:** 10.1016/j.jaapos.2020.08.009

**Published:** 2020-12

**Authors:** Michalis Georgiou, Lisa McAnena, Michel Michaelides, M. Ashwin Reddy

**Affiliations:** a Moorfields Eye Hospital, London, United Kingdom; b UCL Institute of Ophthalmology, University College London, United Kingdom; c Royal London Hospital, Whitechapel, London, United Kingdom

## Abstract

**Purpose:**

To investigate the clinical findings and differential diagnosis of incidental unilateral discoid maculopathy in a case series of children.

**Methods:**

The medical records and retinal imaging of children referred to a single center for flat, well circumscribed, hypopigmented discoid macular lesion in the left eye were reviewed retrospectively.

**Results:**

Three children (age range, 4-11 years; 2 female), with no subjective ophthalmic complaints, were referred for investigation of a flat, well-circumscribed, hypopigmented discoid macular lesion in the left eye. Case 1 had a history of viral mesenteric adenitis, and case 2 had a history of hand, foot, and mouth disease. For case 3, no previous history of systemic viral infection was established. Snellen visual acuity was 20/20 for all 3 children. The lesion was located superior to the fovea for case 1 and centered to the fovea for cases 2 and 3, all in the left eye. In all 3 patients, hyperautofluorescent changes were noted around the edges of the lesion, which was roughly discoid. OCT showed subtle changes of the interdigitation zone and retinal pigment epithelium (RPE) for cases 1 and 2. In case 3 the presence of hyperreflective, hypertrophic tissue at the level of the interdigitation zone and/or the RPE was noted**.**

**Conclusions:**

In these 3 children with subclinical, unilateral discoid maculopathy sharing common features and identified incidentally, previous viral illness may have been causative. These cases may represent resolved unilateral acute idiopathic maculopathy.

In recent years, the number of incidental findings and referrals across all the disciplines of medicine, including ophthalmology, has increased because of screening programs and developments in medical imaging.^1^^,^^2^ The epidemiological data for incidental findings are limited and difficult to collect, because they involve private practice and public hospitals, optometrist, ophthalmologists, and other disciplines, as well as screening programs and routine check-ups. Incidental findings can be benign or malignant, inflammatory, infectious, inherited, or congenital-developmental. We present a case series of 3 children with a benign, unilateral discoid maculopathy, sharing common features, identified incidentally, and referred to a tertiary eye center for further investigation.

## Methods

This study complies with all local laws and the principles of the Declaration of Helsinki and was approved by the Moorfields Eye Hospital Ethics Committee. The patients were examined by medical retina specialists, in Moorfields Eye Hospital, London. All available clinical notes and retinal imaging were reviewed. Further detailed medical history was requested from the caring general practitioners of the children.

## Results

**Fig 1:**
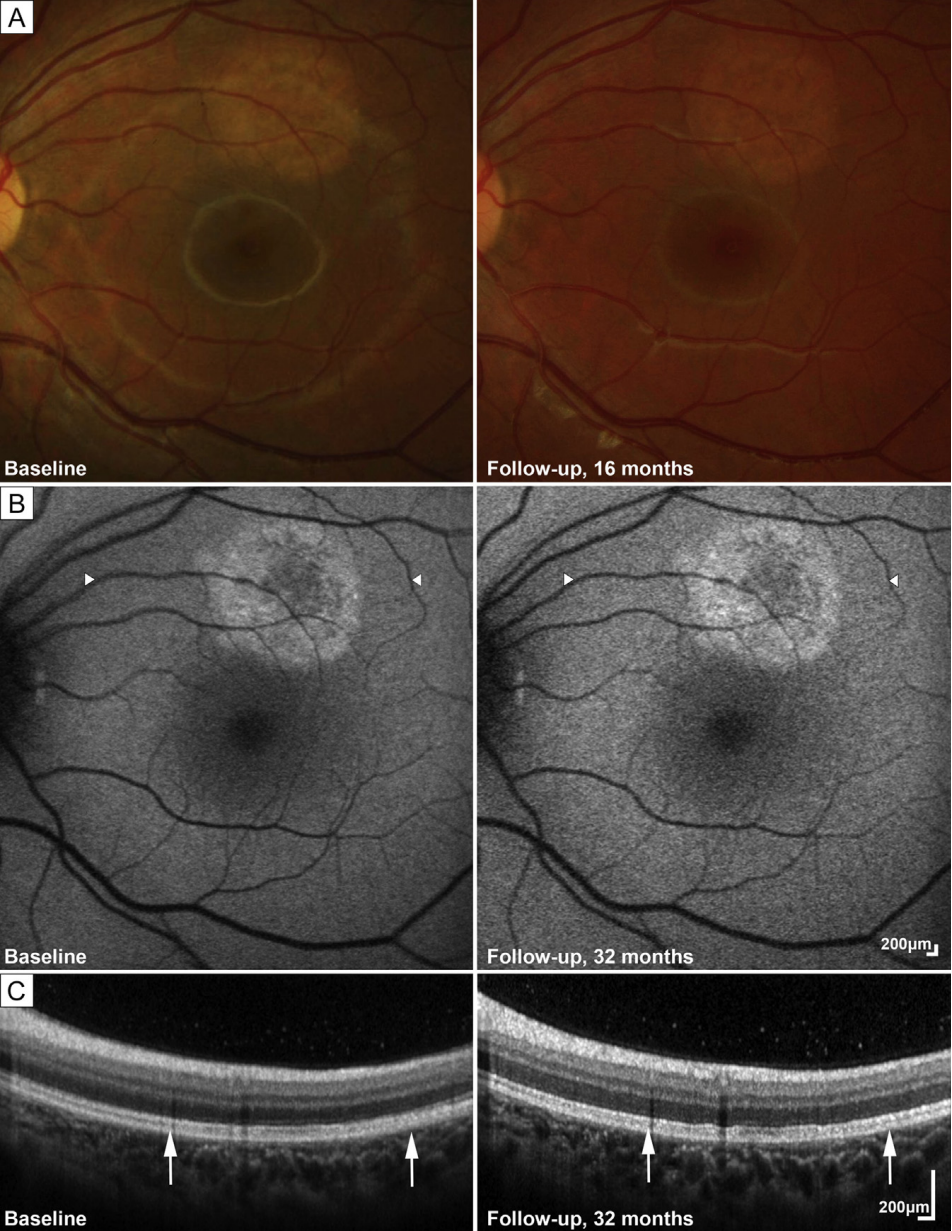
Retinal imaging of case 1. A, Fundus photographs. B, Fundus autofluorescence; arrowheads mark the borders of the lesion in 1C. C, Optical coherence tomography (OCT); arrows mark the borders of the lesion, which was stable over time. The left column presents retinal imaging at baseline examination; the right column, imaging over the follow-up period.

**Fig 2:**
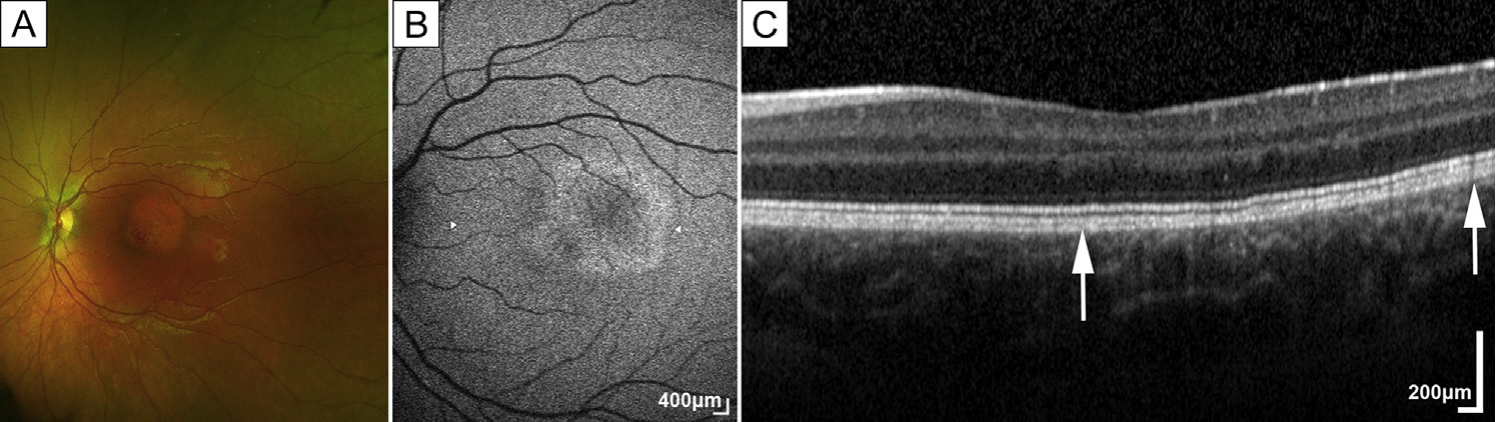
Retinal imaging of case 2. A, Fundus photographs with a central hypopigmented lesion between the fovea and the superior arcade. B, Fundus autofluorescence (arrowheads mark the borders of the lesion in 2C). C, OCT; arrows mark the borders of the lesion.

**Fig 3:**
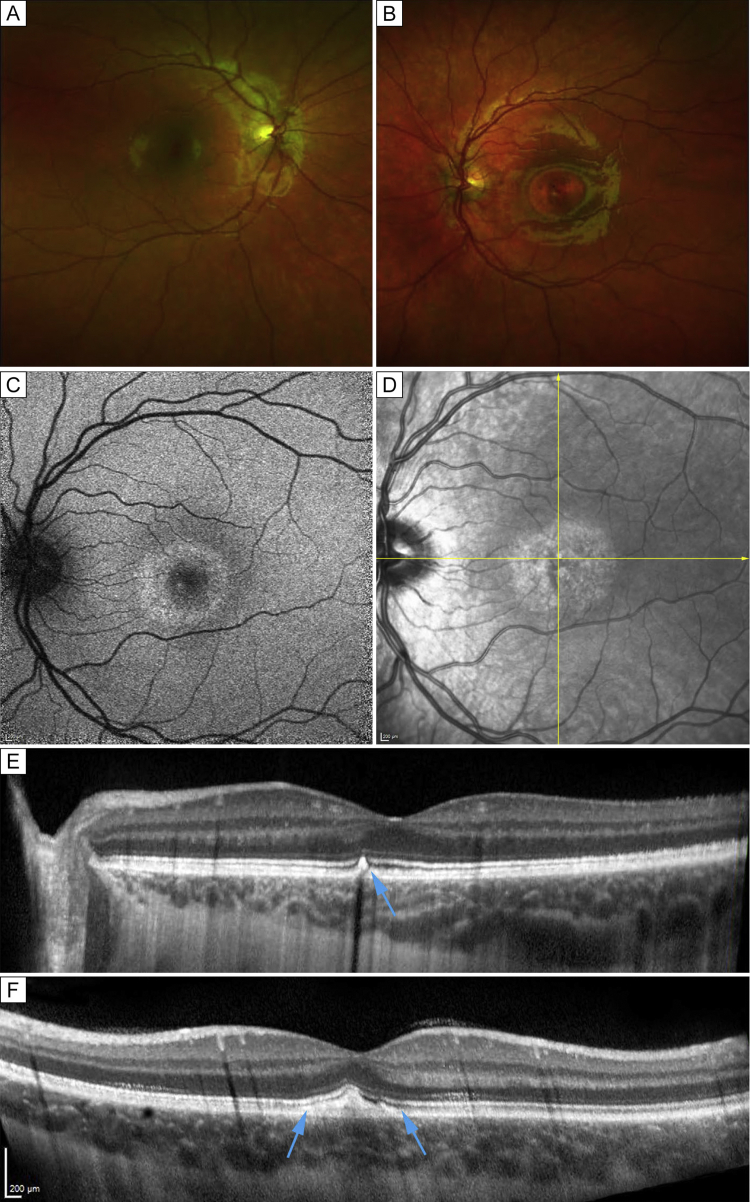
Retinal imaging of case 3. A-B, Fundus photographs of the right (A) and left (B) eyes. A central hypopigmented lesion was observed in the left eye. C, Fundus autofluorescence image showing centrally increased autofluorescence compared with normal foveal hypoautofluorescence, surrounded by a halo of increased autofluorescence. D, Near infrared reflectance fundus image; the arrows mark the horizontal and vertical transfoveal OCT line scans presented in 2E and 2F, respectively. The arrows mark the hypertrophic, hyperreflective structure at the level of the interdigitation zone and the retinal pigment epithelium.

Three children (age range, 4-11 years; 2 female) were referred after presenting to their local optometrist for routine eye examination, with no subjective ophthalmic complaints, on discovery of an unusual left circular macular lesion (Fig 1, Fig 2, Fig 3).

All 3 children denied any visual disturbance and had negative past ophthalmic, recent medical, birth, and family histories, including trauma and congenital infections. On further detailed medical history and direct contact with their general practitioners; case 1 had a history of viral mesenteric adenitis at 8 years of age, and case 2 had a history of hand, foot, and mouth disease (HFMD) at 15 months of age, with both cases having no visual complaints or ophthalmological evaluation at the time of the disease, and both received only supportive care. For case 3, no previous history of systemic viral infection was established other than upper respiratory tract infections.

On examination, uncorrected Snellen visual acuity was 20/20 in both eyes for all 3 children. Anterior segments were normal, with clear vitreous and clear visual axes. The right fundus was normal in all (Figure 3A). Fundus examination of the left eye revealed a flat, well circumscribed, circular, hypopigmented discoid macular lesion in all 3 children. The lesion was approximately 1.5 disk diameters in size and was located superior to the fovea for case 1 (Figure 1) and centered to the fovea in cases 2 (Figure 2) and 3 (Figure 3).

Fundus autofluorescence imaging revealed hyperautofluorescent changes around the edges of the lesion, with the lesion being roughly discoid and similar in size and shape to the hypopigmented area on color fundus photographs (Fig 1, Fig 2, Fig 3). All 3 patients had a similar autofluorescence pattern.

Optical coherence tomography (OCT) revealed showed subtle changes of the interdigitation zone and retinal pigment epithelium (RPE) for cases 1 and 2 (Figures 1 and 2). In case 3, the presence of hyperreflective, hypertrophic tissue at the level of the interdigitation zone and/or the RPE was noted (Figure 3).

Case 1, over 32 months' follow-up, remained asymptomatic, with stable visual acuity, clinical examination, and retinal imaging (Figure 1). The lesion was undetectable on ultrasound; both pattern and full-field electroretinography were normal. Case 2 was clinically stable at 6 months’ follow-up. No follow-up visits were available for case 3.

## Discussion

Despite the appearance of a flat, mottled, hypopigmented lesion in a well-circumscribed shape, the lesions in these 3 cases appeared to have no functional significance at presentation: patients were asymptomatic, with excellent visual acuity. Lesions demonstrated hyperautofluorescence, particularly at the border of the lesion, with corresponding mild OCT changes. The lesions also remained stable in size and appearance over time when evaluated longitudinally (Figure 1).

The differential diagnoses that could be considered in these cases include congenital hypertrophy of the RPE and atypical North Carolina macular dystrophy. It is also possible that the lesions are secondary to an antenatal or perinatal infection. However, these lesions are not in keeping with any of these diagnoses. The inactive stage of acute retinal pigment epithelitis (ARPE, or Krill disease)^3^, ^4^, ^5^ and inactive state of unilateral acute idiopathic maculopathy (UAIM) described by Yannuzzi and colleagues^6^ in 1991 are also possible diagnoses. Both ARPE and UAIM most commonly present with unilateral maculopathy centered to the fovea and *severe* vision loss.^3^^,^^6^^,^^7^ Both conditions have a favorable prognosis of complete resolution in most cases, are rare, and of unknown incidence, affecting usually young healthy individuals with or without the presence of recent viral illness.^3^, ^4^, ^5^, ^6^, ^7^, ^8^ In the acute phase, UAIM and ARPE can be differentiated on OCT: UAIM shows swelling of the outer retina with presence of hyper-reflective exudation and neurosensory retinal detachment,^9^ whereas ARPE shows a dome-shaped hyper-reflective lesion at the photoreceptor outer segment layer,^3^ which occasionally can involve the full thickness of the fovea.^10^ All 3 of our cases presented with inactive disease, and because of the eccentric localization in case 1, the RPE hypertrophy in case 3, the lack of any symptoms of visual disturbance at any time, and the size of all three lesions, they are not entirely typical for UAIM, although they could represent atypical UAIM.

Despite the lack of past ophthalmological history, the past medical history of mesenteric adenitis, HFMD, and upper respiratory viral illness favor UAIM. However, the lesion is outside the fovea in case 1, which would not suggest UAIM; but it is subfoveal in cases 2 and 3, with the child in case 2 being only 15 months of age when he had HFMD, and subnormal visual acuity would likely have been missed. However, the other 2 children also did not present at the possible acute phase of the disorder or have any visual symptoms. Of note, there is little in the literature regarding long-term evaluation of the structural changes in UAIM, probably because of the benign natural history of the disease, with complete resolution in most patients within weeks.^6^^,^^7^ The pathophysiology of UAIM is thought to contain an inflammatory component and it is not fully understood.

In a detailed review of all the available studies on PubMed with at least an abstract in English, retrieved on searching for the terms *unilateral acute idiopathic maculopathy* and UAIM, we identified 67 cases, reported in 23 case reports and 7 case series of 3 to 17 patients. Eccentric localization, as presented in our case 1, was described in 5 of 67 patients with UAIM^7^^,^^11^; bilateral disease was rare (4/67).^7^^,^^12^^,^^13^ In 28 unilateral cases, the affected eye was specified, with an equal number affecting left and right eyes (14 cases). The disease was self-limited in 64 cases (96%), with visual acuity returning within weeks to baseline without intervention. A single case report of recurrent disease was identified.^14^ Therapeutic intervention with sub-Tenon's injection of triamcinolone acetonide was reported in a patient with bilateral disease and anterior uveits,^12^ and in another reported case aflibercept was used.^15^ The course of the disease was almost universally benign, without residual vision defect, or need of treatment.

A complex pattern of macular hypo- and hyperautoflourescence can be seen in active UAIM.^16^ Early stages of the disease usually present with neurosensory detachment of the macula and subfoveal fluid, which resolves over the following weeks.^6^^,^^7^ In 1 case a pseudohypopyon was reported.^17^ OCT can aid the diagnosis by revealing neurosensory detachment and the morphological changes in the RPE and outer retina.^9^^,^^18^ Mottled pigmentation can present in the macula, developing in conjunction with resolution of neurosensory detachment. Abnormal hyper-reflectivity and thickening at the level of the RPE and outer retina in the acute phase and residual subfoveal hyper-reflectivity after resolution was previously reported,^14^^,^^18^^,^^19^ a finding presented in our case 3 (Figure 3).

Acute and convalescent coxsackie virus infection has been previously suggested as causative.^20^ No acute or convalescent viral titers were available for any of our cases. In the literature, the state of health at the time of UAIM or just prior to the ophthalmic manifestation has been clearly documented in 46 cases, with the following findings: flulike illness (n = 13),^6^^,^^21^, ^22^, ^23^, ^24^, ^25^, ^26^ healthy (negative for recent medical history; n = 12),^6^^,^^9^^,^^12^^,^^14^^,^^19^^,^^27^, ^28^, ^29^, ^30^, ^31^, ^32^ HFMD (n = 7),^8^^,^^9^^,^^20^^,^^33^, ^34^, ^35^ pregnancy (n = 3),^1^^,^^7^ orchitis (n = 2),^9^ anterior uveitis (n = 1),^12^ acute tonsillitis (n = 1),^12^ pharyngitis (n = 1),^18^ laryngitis (n = 1),^15^ gastroenteritis (n = 1),^13^ and Hashimoto disease (n = 1).^12^ Other specific viral infections have also been implicated, including HIV (n = 1),^7^ yellow fever (n = 1),^36^ and Zika virus (n = 1).^37^ In 26 cases (57%) it seems that a viral infection was coincident with disease, which is not readily understandable, given the far more common unilateral presentation of UAIM when bilateral disease would be expected. In general health states such as pregnancy, positive HIV status (not acute HIV infection or AIDS), and Hashimoto disease, it is even more unclear how they may relate to UAIM, because they are common in the otherwise healthy young population. In patients presenting in a nonacute stage and who are asymptomatic, screening for viral antibodies has no clinical significance, because positive viral titers are common in the general population, the disease is benign, and many reported cases even in an acute phase were negative for convalescent titers.

The subclinical findings in our 3 patients may be due to UAIM; however, this remains speculative. Only 1 case of an incidental *discoid* lesion in an asymptomatic 15-year-old girl with negative past ophthalmic history has been attributed to UAIM in the literature.^31^ In the largest cohort reported to date (n = 17), the age range was 25-35 years old (without individual ages being reported).^7^ Forty-six reported cases in the literature have an average age of 32.7 ± 11.2 years (range, 14-59). Despite the aforementioned association of viral prodrome and UAIM and viral infections being very common in childhood, no case has been previously reported in young children. This is the first case series of possible UAIM in patients under the age of 14 years and the first cohort to suggest the possible subclinical course of the acute phase of the disease in early childhood.
